# tRNA-derived small RNAs in plant response to biotic and abiotic stresses

**DOI:** 10.3389/fpls.2023.1131977

**Published:** 2023-01-30

**Authors:** Chaojun Wang, Weiqiang Chen, Maimaiti Aili, Lei Zhu, Yan Chen

**Affiliations:** ^1^Institute of Education Science, Leshan Normal University, Leshan, China; ^2^Key Laboratory of Beijing for Identification and Safety Evaluation of Chinese Medicine, Institute of Chinese Materia Medica, China Academy of Chinese Medical Sciences, Beijing, China; ^3^Xinjiang Institute of Traditional Uyghur Medicine, Urumqi, China; ^4^Institute of Thoracic Oncology and Department of Thoracic Surgery, West China Hospital, Sichuan University, Chengdu, China; ^5^Guangzhou Key Laboratory of Crop Gene Editing, Innovative Center of Molecular Genetics and Evolution, School of Life Sciences, Guangzhou University, Guangzhou, China

**Keywords:** plant, tsRNAs, stress response, expression regulation, biological function

## Abstract

tRNA-derived small RNAs (tsRNAs) represent a novel category of small non-coding RNAs and serve as a new regulator of gene expression at both transcriptional and post-transcriptional levels. Growing evidence indicates that tsRNAs can be induced by diverse stimuli and regulate stress-responsive target genes, allowing plants to adapt to unfavorable environments. Here, we discuss the latest developments about the biogenesis and classification of tsRNAs and highlight the expression regulation and potential function of tsRNAs in plant biotic and abiotic stress responses. Of note, we also collect useful bioinformatics tools and resources for tsRNAs study in plants. Finally, we propose current limitations and future directions for plant tsRNAs research. These recent discoveries have refined our understanding of whether and how tsRNAs enhance plant stress tolerance.

## Introduction

Being sessile, plants are continuously exposed to a variety of biotic and abiotic stresses, e.g., salt, drought, cold or heat stress as well as fungal or virus infection, which are major constrains for the growth, productivity and quality of all kinds of agricultural and horticultural plants. To cope with these extreme situations and resist the resulting adverse effects, plants have evolved sophisticated response strategies based on multiple gene regulatory mechanisms, including transcriptional regulation by changing epigenetic modifications (Chang et al., 2020) and post-transcriptional regulation through miRNAs induced gene silencing (Jones-Rhoades et al., 2006). With the rapid development of the next-generation sequencing technologies and bioinformatics approaches, tRNA-derived small RNAs (tsRNAs), first considered as byproducts of tRNAs random degradation, have been characterized in all three kingdoms of life as a new class of regulatory small non-coding RNAs involved in a wide range of biological processes, such as growth, development, diseases as well as stress responses (Maute et al., 2013; Goodarzi et al., 2015; Chen et al., 2016; Kim et al., 2017; Zhu et al., 2018a; Zhu et al., 2018b; Yamasaki et al., 2009; Zhu et al., 2019). tsRNAs, depending on the length, cleavage site and precursor type, can be divided into three major types: 5’ or 3’ tRHs (tRNA-derived halves) derived from the cleavage of mature tRNAs at the anticodon loop, 30-35 nt in length; 5’, 3’ or inter tRFs (tRNA-derived fragments) derived from the cleavage of mature tRNAs at the D and/or TψC loop, 10-30 nt in length; 3’U tRF derived from the cleavage of pre-tRNAs by RNase Z during processing (Zhu et al., 2018a; Zhu et al., 2018b*;* Lyons et al., 2018*;* Zhu et al., 2019*;* Ma et al., 2021a).

Growing evidence shows that the expression of several specific tsRNAs in plant are changed obviously under certain stress conditions like oxidative, drought or heat stress, as well as phosphate (Pi) starvation (Thompson et al., 2008; Hsieh et al., 2009; Wang et al., 2011; Loss-Morais et al., 2013; Hackenberg et al., 2013). Functional analyses have demonstrated that those stress-regulated tsRNAs play vital roles in plant response to both biotic and abiotic stresses, often by regulating the expression of stress-related genes (Asha and Soniya, 2016; Gu et al., 2022; Sun et al., 2022). Therefore, characterizing these stress-responsive tsRNAs and understanding tsRNA-guided stress regulatory networks could provide new ways to enhance stress tolerance in plants, which is of great value in sustainable agricultural and horticultural production. In this review, we comprehensively summarize the current progresses in the diversity, biogenesis and function of tsRNAs in plants, and highlight the expression regulation and potential function of plant tsRNAs in biotic and abiotic stress responses. In addition, we also collect the relevant information about useful bioinformatics tools and resources for tsRNAs study in plants. At present, research into tsRNAs still faces tough challenges as how to accurately and efficiently interfere or quantify their expression and thus interpret their exact functions and mechanisms, which require future efforts to develop new and efficient approaches.

## Roles of ribonucleases in RNA metabolism

As ribonucleases (RNases) are responsible for tsRNAs production, we first give a brief introduction of the types and functions of RNases. Primary transcripts are synthesized by RNA polymerases, while subsequent RNA processing to generate shorter functional RNA species (mRNA, rRNA, tRNA or regulatory RNAs) or degradation to eliminate aberrant RNAs are mainly catalyzed by RNases (Deshpande and Shankar, 2002). RNases are present in almost all organisms including bacteria, virus, yeast, plants, and animals, and play vital roles in RNA metabolism (Irie, 1999). They come in two categories namely endoribonucleases and exoribonucleases on the basis of their mechanism of action (Condon, 2009; Matos et al., 2011). Endoribonucleases cut RNA molecules internally like a pair of scissors while exoribonucleases remove terminal nucleotides from either the 3’ end or the 5’ end of the RNA molecules as a “Pacman”. RNases can act on single-stranded RNAs, double-stranded RNAs and DNA-RNA hybrids hydrolytically or phosphorolytically (Irie, 1999; Condon, 2009; Luhtala and Parker, 2010; Matos et al., 2011).

In plants, the majority of RNases cleave RNAs *via* the formation of 2’,3’-cyclic phosphate (cP) intermediates, ultimately generating oligo- or mononucleotides with a 3’-phosphate group (Eun, 1996; Irie, 1999). The 2’,3’-cyclizing RNases, also known as transferase-type RNases, include three groups corresponding to RNase T1, RNase A and RNase T2 families ( (Irie, 1999; MacIntosh, 2011). These RNases are usually secreted or targeted to organelles associated with the secretory system such as the lysosome or vacuole (Deshpande and Shankar, 2002; MacIntosh, 2011). Thus, they are localized in a space normally without the presence of RNA substrates. Enzymes from RNase T1 family are guanylic acid specific alkaline RNases with optimal pH7-8 and distributed in certain species of fungi and bacteria. The vertebrate-specific RNase A family is weakly acidic (pH6.5-7) or alkaline (pH7-8), with pyrimidine base specificity. First purified from the fungal *Aspergillus orzae*, RNase T2 family proteins are acidic transferase-type endoribonucleases without base-specificity, present in almost all organisms and highly conserved in eukaryotes (Luhtala and Parker, 2010). Phylogenetic analyses have defined three subclasses of the RNase T2 family in plants (MacIntosh and Castandet, 2020). Class I enzymes are diversified, tissue-specific and often regulated by stresses. Class II proteins are highly conserved in plant genomes and carry out a housekeeping role in rRNA recycling, the ancestral function of eukaryotic RNase T2 enzymes. Class III, the first identified plant RNase T2 proteins, were initially cloned in *Nicotiana alata* as self-incompatibility genes (S genes) encoding style-specific glycoproteins, and subsequently shown to be ribonucleases (S-RNases). In *Arabidopsis*, five members of the RNase T2 family have been identified (RNS1-5), among which RNS1, RNS3, RNS4 and RNS5 are categorized as Class I and RNS2 belongs to Class II (MacIntosh et al., 2010). Clearly, understanding the types and mechanisms of RNases are essential for studying the biogenesis and function of different types of RNAs in plant.

## Biogenesis and classification of tsRNAs in plants

**Figure 1:**
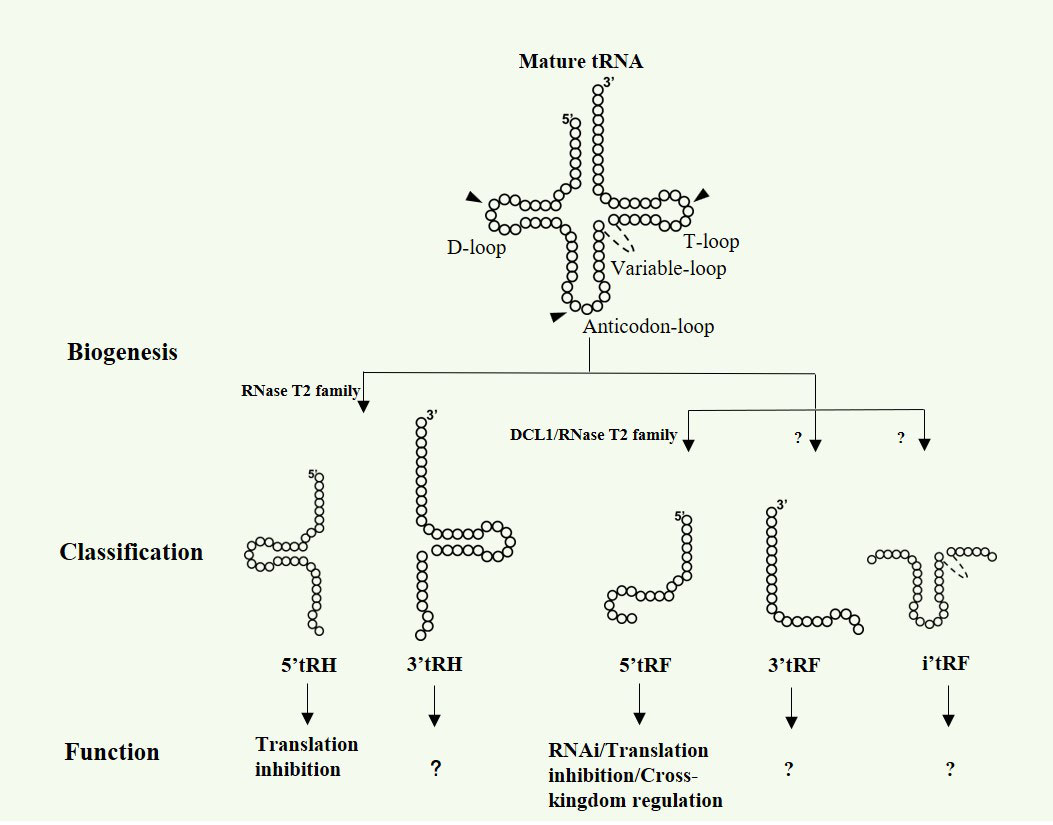
Biogenesis, classification and function of tsRNAs in plants. Arrow heads show the cleavage sites in mature tRNAs, generating 5 types of tsRNAs.

In addition to their well-known function in protein synthesis, tRNAs can be cleaved at specific sites by different endoribonucleases to produce tsRNAs, varying in length, sequence and functions (Zhu et al., 2018a; Zhu et al., 2018b; Ma et al., 2021a). Broadly, tsRNAs can be classified as three main categories: 5’ or 3’ tRHs, 5’, 3’ or inter tRFs and 3’U tRF (Zhu et al., 2018a; Zhu et al., 2018b; Lyons et al., 2018; Zhu et al., 2019; Ma et al., 2021a) (**Figure 1**). Notably, most of the known tsRNAs are derived from mature tRNAs and no 3’U tRFs have been reported in plants so far. In mammals, 5’ or 3’ tRHs are 30-35 nt fragments generated as a result of the tRNA cleavage at the anticodon loop by Angiogenin belonging to the RNase A superfamily (Fu et al., 2009). In *Saccharomyces cerevisiae* and *tetrahymena thermophilus*, tRHs are cleaved by Rny1p and Rnt2, respectively, both of which are from RNase T2 family (Thompson and Parker, 2009; Andersen and Collins, 2012). Recent studies from Megel et al. show that RNase T2, but not Dicer-like proteins (DCLs), are key players for tRHs generation in *Arabidopsis* (Megel et al., 2019). For tRFs biogenesis, it is still somewhat controversial and requires further clarification. Early studies in human HeLa cells reveal that the abundance of a 20 nt tRF derived from tRNA^Gln^ is markedly decreased when the Dicer expression is suppressed by siRNA, indicating the requirement of Dicer for tRFs biogenesis (Cole et al., 2009). However, subsequent small RNA sequencing data show that the mutation of DICER1 does not result in the decrease of tRFs expression in mouse, *Drosophila* and yeast (Kumar et al., 2014). In *Arabidopsis*, Dicer-like 1 (DCL1) is proposed to *be responsible for* the 19 nt tRFs generation in pollen grains (Martinez et al., 2017). Nevertheless, two independent studies indicate that DCLs are not essentially involved in tRFs biogenesis in *Arabidopsis* flower tissue and seedling (Alves et al., 2017; Megel et al., 2019). Megel and collaborators demonstrate that RNS1 and RNS3 are the main endoribonucleases to produce both tRFs and tRHs in siliques, whereas RNS2 is implicated in the tRFs biogenesis in leaf (Megel et al., 2019). Recently, RNS1 and RNS3 are reported to produce 5’tRFs and 5’tRHs from specific mature tRNAs, while the three prime ends of these tsRNAs are *2’,3’-cP*, which further demonstrate the diversity and heterogeny of tsRNAs in plants. Based on the above work, tsRNAs production is quite different from miRNAs that are processed almost exclusively by DCLs. RNase T2 proteins, rather than DCLs, are the main players in plant tsRNAs biogenesis.

## Molecular functions of tsRNAs

Mounting evidence in animals show that tsRNAs are abundantly expressed small non-coding RNAs that can regulate gene expression at multi-dimensional layers, such as transcription inhibition (Zhang et al., 2016), RNA degradation (Maute et al., 2013) and translation regulation (Yamasaki et al., 2009; Ivanov et al., 2011). Although functional studies on plant tsRNAs are relatively limited, a few results show that tsRNAs in plants share similar modes of action with that in animals, such as RNA silencing and translation inhibition (Martinez et al., 2017; Lalande et al., 2020) (**Figure 1**). The functional conservation of tsRNAs between plants and animals could be partially due to their similarity in biogenesis processes, inferring their key role in evolution. Interestingly, cross-kingdom regulation by tsRNAs has also been discovered in plant recently (Ren et al., 2019; Cao et al., 2022) (**Figure 1**).

## RNA interfering

Increasing studies in animals demonstrate that several types of tsRNAs are miRNAs-like for their Dicer-dependent biogenesis and Argonaute (AGO)-associated functional mechanism (Maute et al., 2013; Megel et al., 2019). tsRNAs in animals can recognize RNA targets through sequence complementarity and induce RNA silencing (Maute et al., 2013). However, whether tsRNAs also act in an AGO-dependent manner and involve in the RNAi pathway are still obscure in plants. AGO-associated tRFs were first identified in *Arabiopdsis* by analyzing small RNAs co-immunoprecipitated with AGO proteins, indicating the possible contribution of plant tsRNAs in suppressing gene expression through RNAi pathway (Loss-Morais et al., 2013). Then, based on sequence complementarity between tsRNAs and mRNAs, four possible targets of the AGO-associated tRFs were predicted using a well-known plant small RNA target analysis server, namely psRNATarget (Dai and Zhao, 2011). Further degradome analyses, generally used to identify miRNAs cleavage sites and ta-siRNAs (trans-acting siRNAs) targets, were applied to confirm the possible cleavage of the four predicted tRFs targets, which can lower the false positive rate in the tsRNAs target prediction. Besides, AGO1-immunoprecipitated (AGO1-IP) small RNAs sequencing data from roots and flowers of *Arabidopsis* show that tsRNAs are derived from both nucleus- and plastid-encoded tRNAs. Further analyses of total RNAs and AGO1-IP small RNAs from *Arabidopsis* leaf treated with or without UV reveal that the amounts of 5’ tRF from plastid-encoded tRNAs in total RNAs and/or AGO1-associated small RNAs are both decreased under UV treatment, while a 5’ tRF from nucleus-encoded tRNA^GlyTCC^ is significantly increased in total RNAs and strongly enriched in AGO1, suggesting their potential role in UV stress response (Cognat et al., 2017).

Bioinformatics analyses proved initially that tsRNAs may well associate with AGO system and their potential targets were predicted in several studies. However, standard confirmative experiments, such as Northern blot analyses for AGO-IP small RNAs and target RNA cleavage products, are still lacking. Plant tsRNAs are first indicated to be processed by DCL1 and mediate the target RNA degradation through AGO1 pathway by Martinez et al. (Martinez et al., 2017). A 19 nt pollen enriched 5’ tRF^AlaAGC^ was shown to be decreased in *dcl1* mutant and enriched in AGO1, and the accumulation of this tRF in AGO1-IP small RNAs disappeared in *dcl1* mutant. These results were further confirmed by Northern blot and demonstrate the specific AGO1 loading of this DCL1 generated tRF. In addition, the target cleavage mediated by another 19 nt pollen enriched 5’ tRF^MetCAT^ is dependent almost completely on DCL1 and partially on AGO1, which was verified through 5’ RLM RACE (5’ RNA Ligase-Mediated Rapid Amplification of cDNA Ends) that can capture the degraded products of target RNAs. Moreover, knockdown of 5’ tRF^MetCAT^ with STTM (short tandem target mimic), a method initially designed for miRNAs silencing, can inhibit the target RNA cleavage (Yan et al., 2012). Thus, this study provides strong evidence that tRFs can regulate target gene expression in an AGO-dependent manner. Most recently, another independent study in *Arabidopsis* raise again that the 19 nt 5’ tRF^AlaAGC^ can suppress target gene expression through AGO1 pathway, which was validated by both Northern blot and 5’ RACE (Gu et al., 2022). Using 5’ RLM RACE, the cleavage of predicted tsRNAs targets was also testified in non-model plant organisms, including wheat and black pepper, whereas there is no evidence for the AGO association of these tsRNAs (Asha and Soniya, 2016; Sun et al., 2022).

## Translation inhibition

It is well-known that amino acid charged tRNAs cooperate with rRNA and involve in protein synthesis. Under amino-acids starvation, uncharged tRNAs can suppress protein translation (Phizicky and Hopper, 2010). Intriguingly, increasing evidence in multiple organisms reveal that tsRNAs can repress or promote translation in an AGO-dependent or independent manner. (Ivanov et al., 2011; Kim et al., 2017; Shi et al., 2019). 5’ tRHs, but not 3’ tRHs, can suppress protein translation in human cells, and the terminal oligoguanine motif containing 4 Gs at the five prime end of 5’ tRHs are required for displacing translation initiation factors engaged in both capped and uncapped mRNAs (Yamasaki et al., 2009; Ivanov et al., 2011). On the other hand, a 3’ tRF derived from tRNA^LeuCAG^ was proved to be able to unfold the duplexed RPS mRNAs at the targeting site, thus facilitating ribosome protein biogenesis and enhancing translation (Kim et al., 2017).

In plants, two studies suggest that plant tsRNAs can inhibit protein translation *in vitro*, while the exact mechanisms are still unclear (Zhang et al., 2009; Lalande et al., 2020). Fragments from non-coding RNAs, such as tRNAs, ribosomal RNAs, and spliceosomal RNAs, were found to be present in the phloem of pumpkin, and total RNAs extracted from phloem sap (PS) can suppress translation *in vitro* (Zhang et al., 2009). To prove the translation inhibition effect is caused by tsRNAs in PS, *in vitro* translation assay was performed using tRNA fragments produced from yeast tRNAs, as it is not feasible technically to isolate pure PS tRNA fragments in high amounts. Indeed, protein translation is inhibited by yeast tRNA fragments *in vitro*, while whether PS tRNA fragments are the principal agents of the translation inhibition remain non-conclusive and in controversy. The other study suggest that *Arabidopsis* tRNA fragments can repress translation in an unspecific manner. A series of oligo ribonucleotides mimicking natural tRFs were analyzed and only two, derived from the 5’ ends of tRNA^AlaAGC^ and tRNA^AsnGTT^, can strongly attenuate translation. Unlike the mechanism in human, the G18 and G19 residues of *Arabidopsis* tRF^Ala^, but not the 4 Gs present at the 5’ ends, are essential for the translation inhibition. Furthermore, the 5’ tRF^AlaAGC^ or 5’ tRF^AsnGTT^ needs to associate with polyribosomes to induce translation inhibition, while sequence complementarity between tRFs and mRNAs is not required, suggesting that tRFs may act as general modulation factors of the translation process in plants (Lalande et al., 2020). Nevertheless, more efforts are needed to elucidate the precise mechanisms of tsRNAs in plant translation inhibition.

## Cross-kingdom regulation

Exogenous plant miRNAs were first detected in the serum and plasma of human and animals by Zhang et al. (Zhang et al., 2012). Food-derived MIR168a, a miRNA highly expressed in rice, was further proved to be able to resist gastrointestinal tract and reach the serum and organs like liver, where it inhibits LDLRAP1 (low-density lipoprotein receptor adapter protein 1) expression and eventually suppresses the removal of LDL from the plasma. Since then, increasing studies revealed the cross-kingdom regulation by plant-derived miRNAs, while several negative evidences of miRNAs transference between kingdoms were also reported (Del Pozo-Acebo et al., 2021). Recently, SIDT1 (SID-1 transmembrane family member 1) expressed on gastric pit cells in the stomach was suggested to be required for the absorption of dietary miRNAs, which not only confirmed the phenomenon of cross-kingdom regulation, but also indicated the great potential of plant small RNAs for therapeutic purposes (Chen et al., 2021). Based on this, a 5’ tRF derived from tRNA^HisGUG^ of Chinese yew, namely tRF-T11, was found to display comparable anti-cancer effects with taxol on ovarian cancer cell A2780 and its xenograft animal model (Cao et al., 2022). It was further proved that tRF-T11 can interact with AGO2 to directly target oncogene TRPA1 and suppress its expression through the RNAi pathway in ovarian cancer cells. This study uncovers a novel role of plant-derived tRFs in regulating endogenous cancer-related genes, showing great promise for exploiting natural RNA drugs for therapeutics. There are no data, however, to indicate whether tRF-T11 from Chinese yew can transfer to another kingdom through diet, which may probably be the case given that plant tsRNAs have similar properties with plant miRNAs in some ways. Remarkably, the cross-kingdom communication of tsRNAs was observed between rhizobial and its host soybean. Rhizobial tRFs can transfer to soybean roots and hijack the host RNAi machinery to silence key host genes, thus enhancing nodulation in soybean (Ren et al., 2019).

## Expression of tsRNAs under stresses

tsRNAs, initially reported as tRNA-derived stress-induced small RNAs in different organisms, can be up-regulated under a variety of stresses including oxidative stress (Thompson et al., 2008), heat (Wang et al., 2016) and drought (Hackenberg et al., 2015). Later studies demonstrated that the up-regulation of tsRNAs is not a general effect of all stresses, as only specific tsRNAs are induced under certain stress conditions. These observations further suggested that tsRNAs are not random degradation products, but potential regulators during stress responses. Accumulating data showed that the expression of plant tsRNAs can be regulated by Pi starvation (Hsieh et al., 2009; Hackenberg et al., 2013), heat stress (Wang et al., 2016), UV treatment (Cognat et al., 2017) and fungal infection (Zahra et al., 2021; Gu et al., 2022; Sun et al., 2022), indicating the possible function of these tsRNAs in plant stress responses (**Table 1**).

**Table 1:** List of tsRNAs studies in plant biotic and abiotic stresses.

	Plant Species	Stress	Stress responsive tsRNAs identified by small RNA-seq or Northern blot	Identification method	Confirmation method	Function	Target characterization method	Reference
Abiotic	*Arabidopsis*	Oxidative	5’ tRH^HisGTG,GluCTC,ArgCCT,TrpCCA^	Northern	\	\	\	Thompson et al., 2008
	*Arabidopsis*	Pi starvation	5’ tRF^AspGTC,GlyTCC^	Small RNA-seq	Northern	\	\	Hsieh et al., 2009
	*Brassica rapa*	Heat	5’ tRF^Ala,Gly^	Small RNA-seq	Northern	\	\	Wang et al., 2011
	Barley	Pi starvation	\	Small RNA-seq	\	\	\	Hackenberg et al., 2013
	*Arabidopsis*	Cold, Drought and Salt	5’ tRF^AlaAGC,ArgCCT,ArgTCG,GlyTCC^	Small RNA-seq	\	RNAi	Prediction	Loss-Morais et al., 2013
	Wheat	Heat	3’ tRF^ThrTGT,TyrGTA^, 5’ tRF^SerTGA^	Small RNA-seq	qPCR	\	\	Wang et al., 2016
	Barley	Drought	i’ tRF^ValAAC^	Small RNA-seq	Northern	\	\	Hackenberg et al., 2015
	*Arabidopsis*	Oxidative	5’ tRF^ArgTCG^, 3’ tRF^TyrGTA^	Small RNA-seq	qPCR	\	\	Alves et al., 2017
	*Arabidopsis*	UV	5’ tRF^GlyGCC,GlyTCC,ProTGG,ValAAC^	Small RNA-seq	\	\	\	Cognat et al., 2017
Biotic	Black Pepper	Phytophthora capsici	5′tRF^AlaCGC^	Small RNA-seq	qPCR	RNAi	Prediction, 5’ RLM RACE	Asha and Soniya, 2016
	Tomato	Tomato mosaic virus	\	Small RNA-seq	\	\	\	Zahra et al., 2021
	*Arabidopsis*	Botrytis cinerea	5’tRF^AlaAGC^	Small RNA-seq	Northern	RNAi	Prediction, 5’ RLM RACE, STTM	Gu et al., 2022
	Wheat	Fusarium head blight	\	Small RNA-seq	\	RNAi	Prediction, 5’ RLM RACE	Sun et al., 2022

## Under abiotic stresses

A number of stress-regulated tsRNAs have been identified in different plant species. Northern blot analyses indicated that 5’ tRHs from tRNA^HisGTG^, tRNA^ArgCCT^ and tRNA^TrpGTA^, but not tRNA^TyrGTA^, were induced under oxidative stress in *Arabidopsis*. In addition, tsRNAs can also be up-regulated in yeast and human Hela cells under oxidative stress, implying that the up-regulation of tsRNAs might be a conserved response to oxidative stress (Thompson et al., 2008).

The introduction of the next-generation sequencing technology has enabled high-throughput detection and evaluation of tsRNAs expression in both model and non-model plants under different stress conditions (Zahra et al., 2021). In *Arabidopsis*, a novel peak at 19 nt was uncovered in root, but not in shoot, by deep sequencing of small RNAs responsive to Pi deficiency (Hsieh et al., 2009). Further analyses revealed that the majority of the 19 nt small RNAs are 5’ tRFs originated from tRNA^AspGTC^ and tRNA^GlyTCC^. The percentage of these two types of 5’ tRFs sharply increased under Pi deficiency, which were further verified by Northern blot. Subsequently, small RNAs were profiled in shoot of barley under Pi sufficiency and deficiency conditions. Six nuclear-derived and four chloroplast-derived tsRNAs were significantly up-regulated in Pi-deficient shoot, whereas four nuclear-derived and one chloroplast-derived tsRNAs were down-regulated under the same condition (Hackenberg et al., 2013). However, this study did not provide sequences of these Pi starvation responsive tsRNAs or confirm their expression levels through RT-qPCR or Northern blot, so no conclusion can be drawn about whether or not Pi deficiency responsive tsRNAs were conserved between barley and *Arabidopsis*. Next, drought responsive small RNAs were investigated in barley and results showed that tsRNAs had the tendency to be up-regulated under drought stress. Similarly, sequence information for these drought responsive tsRNAs was not available (Hackenberg et al., 2015).

Also, a series of tsRNAs responsive to heat stress were characterized through small RNA-seq in different plant species including *Arabidopsis*, *Brassica rapa* and wheat (Wang et al., 2011; Wang et al., 2016; Zahra et al., 2021). In *Arabidopsis*, three tsRNAs exhibited dysregulation after 0.5 hour of heat stress, while the number rose to 42 after 6 hours treatment, suggesting that heat stress induced tsRNAs generation is time-dependent (Zahra et al., 2021). In *Brassica rapa*, a variety of heat responsive chloroplast-derived tsRNAs were uncovered (Wang et al., 2011). Consistent with the deep sequencing result, Northern blot analyses indicated that a 29 nt 5’ tRF^Ala^ was declined under heat stress, while a 17 nt 5’ tRF^Ala^ and a 23 nt 5’ tRF^Gly^ are remarkably increased. In wheat seedlings, 292 tsRNAs were significantly increased and 41 are decreased under heat stress. Besides, most of these heat responsive tsRNAs were classified as 3’ tRFs (67%), suggesting that the increased cleavage of tRNAs was preferentially induced at 3’ ends under heat stress. Furthermore, the expression patterns of four tRFs derived from tRNA^ValCAC^, tRNA^ThrTGT^, tRNA^TyrGTA^ and tRNA^SerTGA^ were tested in wheat under heat stress by real-time RT-PCR. Results showed that stRNA0011d (3’ tRF^TyrGTA^) and stRNA0015 (5’ tRF^SerTGA^) were up-regulated by high temperature, which well coincides with the bioinformatics analyses (Wang et al., 2016).

The above studies unveiled that some tsRNAs only respond to specific stresses. Several studies, on the other hand, indicated that certain types of tsRNAs can be induced by different abiotic stresses. For example, in *Arabidopsis*, the 19 nt 5’ tRF^ArgCCT^ can be up-regulated by both drought and oxidative stresses (Alves et al., 2017), and the salt-induced 5’ tRF^GlyGCC^ also increased under UV treatment (Cognat et al., 2017). Moreover, in wheat seedlings, stRNA0011d (3’ tRF^TyrGUA^) was found to respond to heat, salt and drought stresses (Wang et al., 2016). Besides, the 19 nt 5’ tRF^ArgCCT^ induced by drought in *Arabidopsis* displayed no change in rice under drought stress (Alves et al., 2017), suggesting that the stress response of the same tsRNAs may be varied in different plant species.

## Under biotic stresses

The expression pattern of tsRNAs can also be altered under biotic stresses, for example, fungi or virus infection, indicating their potential role in biotic stress response (Asha and Soniya, 2016; Zahra et al., 2021; Gu et al., 2022). To elucidate the functional role of tsRNAs during *Peronosporales capsica* (*P. capsica*) infection, small RNAs in black pepper were systematically analyzed and a 23 nt 5’ tRF^AlaCGC^ was found to be up-regulated in leaf and root of black pepper infected by *P. capsici* (Asha and Soniya, 2016). Fusarium head blight (FHB) that occurs in wheat is a devastating fungal disease caused by *Fusarium graminearum* (*F. graminearum*). Recently, small RNAs from the spikelets of an FHB-susceptible variety Chinese Spring (CS) and an FHB-resistant variety Sumai3 (SM) with *F. graminearum* infection and mock inoculation were analyzed, respectively. As the first report on tRFs response to FHB in wheat, different responsive patterns of tRFs to *F. graminearum* infection were observed between CS and SM. 1249 putative tRFs were identified, among which 15 tRFs were CS-specific and 12 were SM-specific. 39 tRFs were significantly increased in both wheat varieties after *F. graminearum* challenge and only nine tRFs were down-regulated. The expression patterns of tRF^Glu^, tRF^Lys^ and tRF^Thr^, three highly induced tRFs with significantly higher fold changes in CS than in SM, were further validated by stem-loop qRT-PCR. It is worth mentioning that RNase T2 family members were also induced by *F. graminearum* infection, to which the accumulation of tRFs were closely related (Sun et al., 2022). In *Arabidopsis*, 137 5’ tsRNAs were down-regulated and 13 were up-regulated in *Botrytis cinerea* (*B. cinerea*) inoculated plants compared to mock inoculation, suggesting that *B. cinerea* infection led to the down-regulation of a significant proportion of 5’ tsRNAs (Gu et al., 2022). In addition, 757 differentially expressed tsRNAs were characterized in tomato plant subjected to *Tomato Mosaic Virus* (TMV) infection, of which the majority were categorized as 15 nt tRFs (Zahra et al., 2021).

## Potential roles of tsRNAs under stresses

The regulation of tsRNAs expression under various types of abiotic and biotic stresses have been well documented, while their functional roles during stress response are still poorly understood. Given that tsRNAs are similar with miRNAs regarding the length and AGO-association, several studies applied the mechanism and characteristics of target recognition for miRNAs to tsRNAs. Thus, a substantial portion of the current functional studies were based on one assumption that tsRNAs act like miRNAs. These studies can be classified into three groups: 1) Only predict tsRNAs target using miRNAs target prediction tools. 2) Further validate the cleavage site of target RNAs through 5’ RLM RACE. 3) Test the association between tsRNAs-mediated RNAi and AGO system (**Table 1**).

## Under abiotic stresses

Previous studies in mammals and yeast demonstrated that some tsRNAs induced by abiotic stress can suppress protein translation (Yamasaki et al., 2009), whereas it has not been systematically investigated and remains largely unknown in plants. In *Arabidopsis*, drought induced tRFs were substantially enriched in AGO and the targets of these tsRNAs were characterized using psRNATarget coupled with degradome analyses (Addo-Quaye et al., 2008; Dai and Zhao, 2011). Four putative targets for the drought responsive tRFs were identified, which involve in wounding response (AT3G61060.1), protein phosphorylation (AT3G05050.1), photomorphogenesis (AT2G24790.1) and unknown processes (AT3G57280.1), respectively (Loss-Morais et al., 2013). However, further experiments are needed to prove the authenticity of these tsRNAs targets.

## Under biotic stresses

Research on tsRNAs under biotic stresses is relatively less than that under abiotic stresses, but the biological function of tsRNAs under biotic stresses is much better deciphered. In several studies, tsRNAs targets are predicted and further validated through 5’ RLM RACE and/or AGO-IP assay. For example, to reveal the potential role of 5’ tRF^AlaCGC^, which is induced in black pepper during *Phytophthora capsici* infection, two mRNA homologs of NPR1, a key regulator of salicylic acid-dependent gene expression during systemic acquired resistance, were predicted as its putative targets. Moreover, the 5’ tRF^AlaCGC^ mediated cleavage on the target mRNAs was validated by the modified 5’ RLM RACE experiment (Asha and Soniya, 2016).

To reveal the role of tsRNAs induced by *F. graminearum* infection, targets of all identified tRFs were predicted in wheat. Gene ontology enrichment analyses showed that these targets play pivotal roles in stress response, energy metabolism and protein digestion. Furthermore, transcriptome analyses unveiled that the expression levels of the tRFs targets are negatively associated with those of the corresponding tRFs. qRT-PCR was performed to validate the expression of the putative tRFs target genes and the results are highly consistent with the transcriptome data. What’s more, the inhibitory effect of *F. graminearum* induced tRFs on their target genes was confirmed *in vivo* through 5’ RLM RACE (Sun et al., 2022). The above analyses suggested that tRFs induced by *F. graminearum* infection might inhibit the expression of the disease resistance-related targets and consequently contribute host susceptibility to *F. graminearum.*

A recent study in *Arabidopsis* showed that the expression of *CYP71A13* (*At2g30770*), which is involved in camalexin biosynthesis and critical for plant defense against *Botrytis cinerea*, is negatively correlated with that of 5’-tsR-Ala (5’ tRF^AlaAGC^), the most abundant 5’ tsRNAs identified by RtcB sRNA-seq (Gu et al., 2022). Furthermore, 5’-tsR-Ala was detected as the most abundant 5’ tsRNAs in AGO1 immunoprecipitates (IPs). Northern blot analyses confirmed that 5’-tsR-Ala accumulation was significantly decreased in *ago1* mutants, wherein the expression of *CYP71A13* was increased. In addition, the 5’-tsR-Ala mediated cleavage of *CYP71A13* mRNA was proved by 5’ RACE. These findings indicate that 5’-tsR-Ala may function as a miRNA and repress *CYP71A13* expression through associating with AGO1. What’s more, the negative regulation by 5’-tsR-Ala of *CYP71A13* expression and anti-fungal defense was again borne out *in vivo* through knocking down 5’-tsR-Ala using the STTM method. Thus, this study unraveled the important role of a 5’ tRF in regulating anti-fungal defense by modifying gene expression through direct target cleavage.

## Bioinformatics tools and resources for tsRNAs study

### tsRNAs identification pipelines

With the fast development and wide application of high-throughput sequencing technology, a considerable body of small RNA-seq datasets have emerged, covering different biological or pathological processes in various plant species. These publicly available data provide valuable resources for the characterization, expression analysis and functional exploration of tsRNAs. Accordingly, increasing pipelines for tsRNAs characterization are developed (Shi et al., 2018; Zahra et al., 2021; Donovan et al., 2021; Ma et al., 2021b; Rawal et al., 2022), which has greatly facilitated tsRNAs research. For example, SPORTS1.0 is a tool for annotating and profiling non-coding RNAs optimized for rRNA and tRNA derived small RNAs and available for a wide range of 68 species across bacteria, yeast, plant and animal kingdoms (Shi et al., 2018). Afterwards, an improved methodology for predicting miRNAs and tsRNAs in both model and non-model organisms were developed, which have expanded the tsRNAs study in more plants without genome reference (Rawal et al., 2022).

### tsRNAs database

Several plant tsRNAs expression database have been developed, making tsRNAs expression analysis much easier for those researchers without bioinformatic background. tRex is the first on line resource dedicated to tsRNAs in *Arabidopsis thaliana (* http://combio.pl/trex*)*. tRex collates the in-house-generated and publicly available small RNA-seq data from various tissues, ecotypes, genotypes and stress conditions, as well as provides web-based tools for tsRNAs identification, RNA structure analyses, modification predictions and target predictions (Thompson et al., 2018). Later, a plant tsRNAs database named PtRFdb was introduced based on the analyses of 1344 small RNA-seq datasets from 10 different plant species, and 5607 unique tRFs, represented by 487,765 entries, were identified (*http://www.nipgr.res.in/PtRFdb/*). Besides, the information of experimentally identified tsRNAs available in literatures from *Arabidopsis thaliana*, *Medicago truncatula*, *Oryza sativa*, *Piper nigum* and *Triticum aestivum* were collected, which can be downloaded as an excel sheet (Gupta et al., 2018). PtncRNAdb, another plant tsRNAs web resource, consists of 4,809,503 tsRNAs entries identified from ~2500 small RNA-seq libraries generated in six plants including *Arabidopsis thaliana*, *Cicer arietinum*, *Zea mays*, *Oryza sativa*, *Medicago truncatula* and *Solanum lycopersicum (* https://nipgr.ac.in/PtncRNAdb*)*. The ‘DE tncRNAs’ is a feature module in PtncRNAdb for differential expression analysis of tsRNAs under various conditions. Apart from the basic information about tsRNAs, the modification, secondary structure, putative targets, interactive networks of target enrichment and related publications can also be obtained for further interpretation of their biological functions (Zahra et al., 2022). Recently, we developed a comprehensive tsRNAs database named tsRBase. tsRBase covers 20 species and 6 of them are plants, viz., *Arabidopsis thaliana*, *Glycine max*, *Oryza sativa*, *Physcomitrella patens*, *Vitis vinifera* and *Zea mays (* http://www.tsrbase.org*)*. tsRBase not only provides differential expression analysis, but also incorporates experimentally validated targets of tsRNAs (Zuo et al., 2021).

### Target prediction tools

Target identification is central for defining the biological function of tsRNAs, whereas it is unrealistic to characterize the targets for all tsRNAs experimentally and there have been few studies on the relationship between tsRNAs and mRNAs, especially in plants. Therefore, researchers have to predict the targets based on algorithms. miRNAs target prediction tools, such as psRNATarget and PsRobot, have been broadly applied to predict tsRNAs targets given that tsRNAs may also suppress gene expression through sequence complementarity (Addo-Quaye et al., 2008; Megel et al., 2019; Zahra et al., 2021). Several computation tools have also been developed specifically for predicting tsRNAs targets in mammals, including tRFTars, tRFTar and tRForest. tRFTars is the first database for tsRNAs target prediction *(* http://trftars.cmuzhenninglab.org:3838/tar/*)*. First, features that influence tsRNAs targeting were screened. Then, tsRNA-mRNA pairs identified by crosslinking, ligation and sequencing of hybrids (CLASH) and covalent ligation of endogenous AGO-bound RNAs (CLEAR)-CLIP were used to select key features through a genetic algorithm (GA). Finally, support vector machine (SVM) was applied to construct tsRNAs prediction models with the selected key features (Shi et al., 2018). tRFTar is a resource for predicting tRF target gene interactions (TGIs) based on the fact that tsRNAs can be loaded onto AGO family proteins to perform post-transcriptional regulations (http://www.rnanut.net/tRFTar/). 146 cross-linking immunoprecipitation and high-throughput sequencing (CLIP-seq) datasets were systematically reanalyzed and 920,690 TGIs between 12,102 tRFs and 5,688 target genes were identified. tRFTar enables various functions like custom searching, co-expressed TGI filtering, genome browser and TGI-based tRF functional enrichment analysis (Rawal et al., 2022). Recently, using cross-linking, Ligation, and Sequencing of Hybrids (CLASH) data as the training and testing dataset, a novel tsRNAs target prediction tool, tRForest, was developed based on the random forest machine learning algorithm (Parikh et al., 2022) *(* https://trforest.com*)*. However, no specific target prediction tools are currently available for plant tsRNAs, so the development of tsRNAs target prediction tools is an urgent issue for plant tsRNAs study.

## Conclusion and future perspective

With the help of improved high-throughput sequencing technologies, a large body of tsRNAs have been identified in various organisms and the numbers are still expanding (Zhu et al., 2018a; Zhu et al., 2018b; Zuo et al., 2021). tsRNAs are thought as a heterogeneous class of small RNAs because of their multitudinous sources and lengths (Zuo et al., 2021). The spatially and temporally regulated expression pattern of tsRNAs has been proposed to play important roles in plant development and stress response. However, direct and in-depth functional analyses of tsRNAs are still missing, especially in plants. Conventional methods for dissecting gene function relied much on genetic mutants. However, this approach is not feasible for the study of tsRNAs due to their small sizes, non-coding property, multiple members and overlapping sequences with tRNAs that is indispensable for protein translation and normal cellular processes. In fact, relevant technologies and achievements regarding tsRNAs study in plants still lag far behind those in animals. Antisense oligonucleotides (ASOs) are widely applied to specifically bind target tsRNAs in mammalian cells (Goodarzi et al., 2015; Kim et al., 2017), which can efficiently knock-down the abundance of corresponding tsRNAs and testify their involvement or functional role in certain physiological and pathological processes more straightforwardly, thus offering a promising alternative to therapies. In plants, there are piecemeal applications of STTM for tsRNAs block (Martinez et al., 2017; Gu et al., 2022). As is known, miRNAs are generally 21 nt in length, and the three-nucleotide bulge that prevent the cleavage of the target mimic (TM) stuck out between the 10th and 11th nucleotide of the targeted miRNAs (Yan et al., 2012). Therefore, it remains to be seen whether STTM is applicable or just as efficient for tsRNAs with other lengths. Besides, results in different organisms showed that specific tsRNAs are associated with protein translation machinery or AGO system and regulate gene expression post-transcriptionally (Ivanov et al., 2011; Maute et al., 2013). In mammals, methods to identify tsRNAs associated proteins have been applied, which allows a more comprehensive exploration of the mechanism and characteristics of tsRNAs (Keam et al., 2014; Goodarzi et al., 2015; Cho et al., 2019). Further efforts are needed to develop new methods for characterizing tsRNAs associated proteins in plants.

Another point worth noting is that traditional small RNAs cloning methods applied by most studies can only capture those with 5’-OH and 3’-Pi. Actually, a large proportion of tsRNAs generated by endoribonucleases are ended with 2’,3’-cP, so they cannot be ligated to the adaptors directly (Shi et al., 2021; Gu et al., 2022). Other internal modifications embedded in tsRNAs, such as methylation, will suppress the reverse transcription and consequently impact the cloning efficiency. Recently, several studies have improved the small RNA cloning methods through removing the end and internal modifications present in small RNAs, which will greatly benefit the tsRNAs research in plants (Shi et al., 2021; Wang et al., 2021; Gu et al., 2022).

## Author contributions

Conceptualization: YC and LZ. Data curation: LZ and YC. Original draft preparation: CW, WC and MA. Supervision: YC and LZ. All authors contributed to the article and approved the submitted version.

## References

[B1] Addo-Quaye C. Eshoo T. W. Bartel D. P. Axtell M. J. (2008). Endogenous siRNA and miRNA targets identified by sequencing of the *Arabidopsis* degradome. Curr. Biol. 18 (10), 758–762. doi: 10.1016/j.cub.2008.04.042 18472421 PMC2583427

[B2] Alves C. S. Vicentini R. Duarte G. T. Pinoti V. F. Vincentz M. Nogueira F. T. (2017). Genome-wide identification and characterization of tRNA-derived RNA fragments in land plants. Plant Mol. Biol. 93 (1-2), 35–48. doi: 10.1007/s11103-016-0545-9 27681945

[B3] Andersen K. L. Collins K. (2012). Several RNase T2 enzymes function in induced tRNA and rRNA turnover in the ciliate *Tetrahymena* . Mol. Biol. Cell 23 (1), 36–44. doi: 10.1091/mbc.E11-08-0689 22049026 PMC3248902

[B4] Asha S. Soniya E. V. (2016). Transfer RNA derived small RNAs targeting defense responsive genes are induced during *Phytophthora capsici* infection in black pepper (*Piper nigrum* l.). Front. Plant Sci. doi: 10.3389/fpls.2016.00767 PMC4887504 27313593

[B5] Cao K. Y. Yan T. M. Zhang J. Z. Chan T. F. Li J. Li C.. (2022). A tRNA-derived fragment from Chinese yew suppresses ovarian cancer growth *via* targeting TRPA1. Mol. Ther. Nucleic Acids 27, 718–732. doi: 10.1016/j.omtn.2021.12.037 35317282 PMC8905250

[B6] Chang Y. N. Zhu C. Jiang J. Zhang H. Zhu J. K. Duan C. G. (2020). Epigenetic regulation in plant abiotic stress responses. J. Integr. Plant Biol. 62 (5), 563–580. doi: 10.1111/jipb.12901 31872527

[B7] Chen Q. Yan M. Cao Z. Li X. Zhang Y. Shi J.. (2016). Sperm tsRNAs contribute to intergenerational inheritance of an acquired metabolic disorder. Science. 351 (6271), 397–400. doi: 10.1126/science.aad7977 26721680

[B8] Chen Q. Zhang F. Dong L. Wu H. Xu J. Li H.. (2021). SIDT1-dependent absorption in the stomach mediates host uptake of dietary and orally administered microRNAs. Cell Res. 31 (3), 247–258. doi: 10.1038/s41422-020-0389-3 32801357 PMC8026584

[B9] Cho H. Lee W. Kim G. W. Lee S. H. Moon J. S. Kim M.. (2019). Regulation of La/SSB-dependent viral gene expression by pre-tRNA 3' trailer-derived tRNA fragments. Nucleic Acids Res. 47 (18), 9888–9901. doi: 10.1093/nar/gkz732 31504775 PMC6765225

[B10] Cognat V. Morelle G. Megel C. Lalande S. Molinier J. Vincent T.. (2017). The nuclear and organellar tRNA-derived RNA fragment population in *Arabidopsis thaliana* is highly dynamic. Nucleic Acids Res. 45, 3460–3472. doi: 10.1093/nar/gkw1122 27899576 PMC5389709

[B11] Cole C. Sobala A. Lu C. Thatcher S. R. Bowman A. Brown J. W.. (2009). Filtering of deep sequencing data reveals the existence of abundant dicer-dependent small RNAs derived from tRNAs. RNA. 15 (12), 2147–2160. doi: 10.1261/rna.1738409 19850906 PMC2779667

[B12] Condon C. (2009). “RNA Processing,” in Encyclopedia of microbiology (Third edition). Ed. Schaechter M. (Oxford: Academic Press), 395–408.

[B13] Dai X. Zhao P. X. (2011). psRNATarget: A plant small RNA target analysis server. Nucleic Acids Res. 39, W155–W159. doi: 10.1093/nar/gkr319 21622958 PMC3125753

[B14] Del Pozo-Acebo L. Lopez de Las Hazas M. C. Margolles A. Davalos A. Garcia-Ruiz A. (2021). Eating microRNAs: Pharmacological opportunities for cross-kingdom regulation and implications in host gene and gut microbiota modulation. Br. J. Pharmacol. 178 (11), 2218–2245. doi: 10.1111/bph.15421 33644849

[B15] Deshpande R. A. Shankar V. (2002). Ribonucleases from T2 family. Crit. Rev. Microbiol. 28 (2), 79–122. doi: 10.1080/1040-840291046704 12109772

[B16] Donovan P. D. McHale N. M. Veno M. T. Prehn J. H. M. (2021). A pipeline for the identification of tRNA and ncRNA fragments from small RNA sequencing data. Bioinformatics 2021:btab515. doi: 10.1093/bioinformatics/btab515 34255836

[B17] Eun H.-M. (1996). “3-nucleases,” in Enzymology primer for recombinant DNA technology. Ed. Eun H.-M. (San Diego: Academic Press), 145–232.

[B18] Fu H. Feng J. Liu Q. Sun F. Tie Y. Zhu J.. (2009). Stress induces tRNA cleavage by angiogenin in mammalian cells. FEBS Lett. 583 (2), 437–442. doi: 10.1016/j.febslet.2008.12.043 19114040

[B19] Goodarzi H. Liu X. Nguyen H. C. Zhang S. Fish L. Tavazoie S. F. (2015). Endogenous tRNA-derived fragments suppress breast cancer progression *via* YBX1 displacement. Cell. 161 (4), 790–802. doi: 10.1016/j.cell.2015.02.053 25957686 PMC4457382

[B20] Gu H. Lian B. Yuan Y. Kong C. Li Y. Liu C.. (2022). A 5' tRNA-ala-derived small RNA regulates anti-fungal defense in plants. Sci. China Life Sci. 65 (1), 1–15. doi: 10.1007/s11427-021-2017-1 34705222

[B21] Gupta N. Singh A. Zahra S. Kumar S. (2018). PtRFdb: A database for plant transfer RNA-derived fragments. Database (Oxford) 2018:bay063. doi: 10.1093/database/bay063 29939244 PMC6016605

[B22] Hackenberg M. Gustafson P. Langridge P. Shi B. J. (2015). Differential expression of microRNAs and other small RNAs in barley between water and drought conditions. Plant Biotechnol. J. 13 (1), 2–13. doi: 10.1111/pbi.12220 24975557 PMC4309496

[B23] Hackenberg M. Huang P. J. Huang C. Y. Shi B. J. Gustafson P. Langridge P. (2013). A comprehensive expression profile of microRNAs and other classes of non-coding small RNAs in barley under phosphorous-deficient and -sufficient conditions. DNA Res. 20 (2), 109–125. doi: 10.1093/dnares/dss037 23266877 PMC3628442

[B24] Hsieh L. C. Lin S. I. Shih A. C. Chen J. W. Lin W. Y. Tseng C. Y.. (2009). Uncovering small RNA-mediated responses to phosphate deficiency in *Arabidopsis* by deep sequencing. Plant Physiol. 151 (4), 2120–2132. doi: 10.1104/pp.109.147280 19854858 PMC2785986

[B25] Irie M. (1999). Structure-function relationships of acid ribonucleases: Lysosomal, vacuolar, and periplasmic enzymes. Pharmacol. Ther. 81 (2), 77–89. doi: 10.1016/s0163-7258(98)00035-7 10190580

[B26] Ivanov P. Emara M. M. Villen J. Gygi S. P. Anderson P. (2011). Angiogenin-induced tRNA fragments inhibit translation initiation. Mol. Cell. 243 (4), 613–623. doi: 10.1016/j.molcel.2011.06.022 PMC3160621 21855800

[B27] Jones-Rhoades M. W. Bartel D. P. Bartel B. (2006). MicroRNAS and their regulatory roles in plants. Annu. Rev. Plant Biol. 57, 19–53. doi: 10.1146/annurev.arplant.57.032905.105218 16669754

[B28] Keam S. P. Young P. E. McCorkindale A. L. Dang T. H. Clancy J. L. Humphreys D. T.. (2014). The human piwi protein Hiwi2 associates with tRNA-derived piRNAs in somatic cells. Nucleic Acids Res. 42 (14), 8984–8995. doi: 10.1093/nar/gku620 25038252 PMC4132735

[B29] Kim H. K. Fuchs G. Wang S. Wei W. Zhang Y. Park H.. (2017). A transfer-RNA-derived small RNA regulates ribosome biogenesis. Nature. 552 (7683), 57–62. doi: 10.1038/nature25005 29186115 PMC6066594

[B30] Kumar P. Anaya J. Mudunuri S. B. Dutta A. (2014). Meta-analysis of tRNA derived RNA fragments reveals that they are evolutionarily conserved and associate with AGO proteins to recognize specific RNA targets. BMC Biol. 12, 78. doi: 10.1186/s12915-014-0078-0 25270025 PMC4203973

[B31] Lalande S. Merret R. Salinas-Giege T. Drouard L. (2020). *Arabidopsis* tRNA-derived fragments as potential modulators of translation. RNA Biol. 17 (8), 1137–1148. doi: 10.1080/15476286.2020.1722514 31994438 PMC7549631

[B32] Loss-Morais G. Waterhouse P. M. Margis R. (2013). Description of plant tRNA-derived RNA fragments (tRFs) associated with argonaute and identification of their putative targets. Biol. Direct. 12 8, 6. doi: 10.1186/1745-6150-8-6 PMC3574835 23402430

[B33] Luhtala N. Parker R. (2010). T2 family ribonucleases: Ancient enzymes with diverse roles. Trends Biochem. Sci. 35 (5), 253–259. doi: 10.1016/j.tibs.2010.02.002 20189811 PMC2888479

[B34] Lyons S. M. Fay M. M. Ivanov P. (2018). The role of RNA modifications in the regulation of tRNA cleavage. FEBS Lett. 592 (17), 2828–2844. doi: 10.1002/1873-3468.13205 30058219 PMC6986807

[B35] MacIntosh G. C. (2011). “RNase T2 family: Enzymatic properties, functional diversity, and evolution of ancient ribonucleases,” in Ribonucleases. Ed. Nicholson A. W. (Berlin Heidelberg: Springer), 89–114.

[B36] MacIntosh G. C. Castandet B. (2020). Organellar and secretory ribonucleases: Major players in plant RNA homeostasis. Plant Physiol. 183 (4), 1438–1452. doi: 10.1104/pp.20.00076 32513833 PMC7401137

[B37] MacIntosh G. C. Hillwig M. S. Meyer A. Flagel L. (2010). RNase T2 genes from rice and the evolution of secretory ribonucleases in plants. Mol. Genet. Genomics 283 (4), 381–396. doi: 10.1007/s00438-010-0524-9 20182746

[B38] Ma X. Liu C. Cao X. (2021a). Plant transfer RNA-derived fragments: Biogenesis and functions. J. Integr. Plant Biol. 63 (8), 1399–1409. doi: 10.1111/jipb.13143 34114725

[B39] Ma X. Liu C. Kong X. Liu J. Zhang S. Liang S.. (2021b). Extensive profiling of the expressions of tRNAs and tRNA-derived fragments (tRFs) reveals the complexities of tRNA and tRF populations in plants. Sci. China Life Sci. 64 (4), 495–511. doi: 10.1007/s11427-020-1891-8 33569675

[B40] Martinez G. Choudury S. G. Slotkin R. K. (2017). tRNA-derived small RNAs target transposable element transcripts. Nucleic Acids Res. 45 (9), 5142–5152. doi: 10.1093/nar/gkx103 28335016 PMC5605234

[B41] Matos R. G. Pobre V. Reis F. P. Andrade J. M. Arraiano C. M. (2011). “Structure and degradation mechanisms of 3′ to 5′ exoribonucleases,” in Ribonucleases,Nucleic acids and molecular biology. Ed. Nicholson A. W. (Berlin, Heidelberg: Springer). doi: 10.1007/978-3-642-21078-5_8

[B42] Maute R. L. Schneider C. Sumazin P. Holmes A. Califano A. Basso K.. (2013). tRNA-derived microRNA modulates proliferation and the DNA damage response and is down-regulated in b cell lymphoma. Proc. Natl. Acad. Sci. U. S. A. 110 (4), 1404–1409. doi: 10.1073/pnas.1206761110 23297232 PMC3557069

[B43] Megel C. Hummel G. Lalande S. Ubrig E. Cognat V. Morelle G.. (2019). Plant RNases T2, but not dicer-like proteins, are major players of tRNA-derived fragments biogenesis. Nucleic Acids Res. 47 (2), 941–952. doi: 10.1093/nar/gky1156 30462257 PMC6344867

[B44] Parikh R. Wilson B. Marrah L. Su Z. Saha S. Kumar P.. (2022). tRForest: a novel random forest-based algorithm for tRNA-derived fragment target prediction. NAR Genom Bioinform. 4 (2), lqac037. doi: 10.1093/nargab/lqac037 35664803 PMC9155213

[B45] Phizicky E. M. Hopper A. K. (2010). tRNA biology charges to the front. Genes Dev. 24 (17), 1832–1860. doi: 10.1101/gad.1956510 20810645 PMC2932967

[B46] Rawal H. C. Ali S. Mondal T. K. (2022). miRPreM and tiRPreM: Improved methodologies for the prediction of miRNAs and tRNA-induced small non-coding RNAs for model and non-model organisms. Brief Bioinform. 23 (1):bbab448. doi: 10.1093/bib/bbab448 34734232

[B47] Ren B. Wang X. Duan J. Ma J. (2019). Rhizobial tRNA-derived small RNAs are signal molecules regulating plant nodulation. Science 365 (6456), 919–922. doi: 10.1126/science.aav8907 31346137

[B48] Shi J. Ko E. A. Sanders K. M. Chen Q. Zhou T. (2018). SPORTS1.0: A tool for annotating and profiling non-coding RNAs optimized for rRNA- and tRNA-derived small RNAs. Genomics Proteomics Bioinf. 16 (2), 144–151. doi: 10.1016/j.gpb.2018.04.004 PMC6112344 29730207

[B49] Shi J. Zhang Y. Tan D. Zhang X. Yan M. Zhang Y.. (2021). PANDORA-seq expands the repertoire of regulatory small RNAs by overcoming RNA modifications. Nat. Cell Biol. 23 (4), 424–436. doi: 10.1038/s41556-021-00652-7 33820973 PMC8236090

[B50] Shi J. Zhang Y. Zhou T. Chen Q. (2019). tsRNAs: The Swiss army knife for translational regulation. Trends Biochem. Sci. 44 (3), 185–189. doi: 10.1016/j.tibs.2018.09.007 30297206 PMC6379142

[B51] Sun Z. Hu Y. Zhou Y. Jiang N. Hu S. Li L.. (2022). tRNA-derived fragments from wheat are potentially involved in susceptibility to fusarium head blight. BMC Plant Biol. 22 (1), 3. doi: 10.1186/s12870-021-03393-9 34979923 PMC8722339

[B52] Thompson D. M. Lu C. Green P. J. Parker R. (2008). tRNA cleavage is a conserved response to oxidative stress in eukaryotes. RNA. 14 (10), 2095–2103. doi: 10.1261/rna.1232808 18719243 PMC2553748

[B53] Thompson D. M. Parker R. (2009). The RNase Rny1p cleaves tRNAs and promotes cell death during oxidative stress in *Saccharomyces cerevisiae* . J. Cell Biol. 185 (1), 43–50. doi: 10.1083/jcb.200811119 19332891 PMC2700514

[B54] Thompson A. Zielezinski A. Plewka P. Szymanski M. Nuc P. Szweykowska-Kulinska Z.. (2018). tRex: a web portal for exploration of tRNA-derived fragments in *Arabidopsis thaliana* . Plant Cell Physiol. 59 (1):e1. doi: 10.1093/pcp/pcx173 29145635

[B55] Wang H. Huang R. Li L. Zhu J. Li Z. Peng C.. (2021). CPA-Seq reveals small ncRNAs with methylated nucleosides and diverse termini. Cell Discov. 7 (1), 25. doi: 10.1038/s41421-021-00265-2 33867522 PMC8053708

[B56] Wang Y. Li H. Sun Q. Yao Y. (2016). Characterization of small RNAs derived from tRNAs, rRNAs and snoRNAs and their response to heat stress in wheat seedlings. PloS One 11 (3):e0150933. doi: 10.1371/journal.pone.0150933 26963812 PMC4786338

[B57] Wang L. Yu X. Wang H. Lu Y. Z. de Ruiter M. Prins M.. (2011). A novel class of heat-responsive small RNAs derived from the chloroplast genome of Chinese cabbage (*Brassica rapa*). BMC Genomics 12, 289. doi: 10.1186/1471-2164-12-289 21639890 PMC3126784

[B58] Yamasaki S. Ivanov P. Hu G. F. Anderson P. (2009). Angiogenin cleaves tRNA and promotes stress-induced translational repression. J. Cell Biol. 185 (1), 35–42. doi: 10.1083/jcb.200811106 19332886 PMC2700517

[B59] Yan J. Gu Y. Jia X. Kang W. Pan S. Tang X.. (2012). Effective small RNA destruction by the expression of a short tandem target mimic in arabidopsis. Plant Cell. 24 (2), 415–427. doi: 10.1105/tpc.111.094144 22345490 PMC3315224

[B60] Zahra S. Bhardwaj R. Sharma S. Singh A. Kumar S. (2022). PtncRNAdb: plant transfer RNA-derived non-coding RNAs (tncRNAs) database. 3 Biotech. 12 (5), 105. doi: 10.1007/s13205-022-03174-7 PMC8986922 35462956

[B61] Zahra S. Singh A. Poddar N. Kumar S. (2021). Transfer RNA-derived non-coding RNAs (tncRNAs): Hidden regulation of plants' transcriptional regulatory circuits. Comput. Struct. Biotechnol. J. 19, 5278–5291. doi: 10.1016/j.csbj.2021.09.021 34630945 PMC8482286

[B62] Zhang X. He X. Liu C. Liu J. Hu Q. Pan T.. (2016). IL-4 inhibits the biogenesis of an epigenetically suppressive PIWI-interacting RNA to upregulate CD1a molecules on monocytes/dendritic cells. J. Immunol. 196 (4), 1591–1603. doi: 10.4049/jimmunol.1500805 26755820

[B63] Zhang L. Hou D. Chen X. Li D. Zhu L. Zhang Y.. (2012). Exogenous plant MIR168a specifically targets mammalian LDLRAP1: evidence of cross-kingdom regulation by microRNA. Cell Res. 22 (1), 107–126. doi: 10.1038/cr.2011.158 21931358 PMC3351925

[B64] Zhang S. Sun L. Kragler F. (2009). The phloem-delivered RNA pool contains small noncoding RNAs and interferes with translation. Plant Physiol. 150 (1), 378–387. doi: 10.1104/pp.108.134767 19261735 PMC2675743

[B65] Zhu L. Li J. Gong Y. Wu Q. Tan S. Sun D.. (2019). Exosomal tRNA-derived small RNA as a promising biomarker for cancer diagnosis. Mol. Cancer. 18 (1), 74. doi: 10.1186/s12943-019-1000-8 30940133 PMC6444574

[B66] Zhu L. Liu X. Pu W. Peng Y. (2018a). tRNA-derived small non-coding RNAs in human disease. Cancer Lett. 419, 1–7. doi: 10.1016/j.canlet.2018.01.015 29337107

[B67] Zhu L. Ow D. W. Dong Z. (2018b). Transfer RNA-derived small RNAs in plants. Sci. China Life Sci. 61 (2), 155–161. doi: 10.1007/s11427-017-9167-5 29170889

[B68] Zuo Y. Zhu L. Guo Z. Liu W. Zhang J. Zeng Z.. (2021). tsRBase: a comprehensive database for expression and function of tsRNAs in multiple species. Nucleic Acids Res. 49 (D1), D1038–D1D45. doi: 10.1093/nar/gkaa888 33068436 PMC7778983

